# Human Papillomavirus in Oral Lichen Planus: Is There an Association? A Meta-Analysis

**DOI:** 10.3390/jcm13133698

**Published:** 2024-06-25

**Authors:** Zuzanna Ślebioda, Tomasz Woźniak, Marzena Liliana Wyganowska

**Affiliations:** 1 Department of Dental Surgery, Periodontology and Oral Mucosa Diseases, Poznań University of Medical Sciences, 61-701 Poznań, Poland; 2 Institute of Human Genetics, Polish Academy of Sciences, 60-479 Poznań, Poland

**Keywords:** human papilloma virus, oral lichen planus, oral mucosa

## Abstract

Lichen planus (LP) is a chronic, recurrent mucocutaneous inflammatory disease with unclearly defined etiology, where a potential role of several viruses has been considered. This meta-analysis aimed to determine the potential association between HPV and oral LP based on case-control and cross-sectional study results. A systematic search was performed in PubMed, Web of Science (SCI), Google Scholar, and Scopus databases with the last update on 6 March 2024. Pooled data were analyzed by calculating odds ratios (OR) and 95% confidence intervals (CI) with the metafor package for R. A total of 13 studies on 541 cases and 413 controls were included in this meta-analysis. It covered eight countries: India, Iran, Turkey, Czech Republic, Hungary, Italy, Macedonia, and the UK. In seven papers, the differentiation into the erosive-atrophic type and non-erosive-atrophic type of OLP was utilized. HPV infection was associated with at least a two times higher risk for a person with HPV to have OLP, depending on whether original data or filled/trimmed data were used. The OR values were 3.54 [2.01, 6.24] and 2.10 [1.16, 3.82], respectively. This meta-analysis was performed to identify the association between HPV and OLP and revealed that HPV infection was associated with at least a high risk for a person with HPV to develop OLP.

## 1. Introduction

Lichen planus (LP) is a chronic, recurrent mucocutaneous inflammatory disease characterized by epithelial thickening or atrophy with possible ulcerations [1,2]. The disturbed immunological response develops and is triggered by several endogenous and exogenous factors. The leading phenomenon is the increased production of T-helper 1 cytokines. Although the etiology of LP is not clearly defined, it is generally accepted to be a T-cell-mediated condition, where the reaction of specific CD8+ T-cells is comparable to the response induced by viruses, which may act as cytoplasmic antigens and induce the expression of host cell proteins. The immunological cascade in LP may be started by an antigen exposing the basal keratinocytes of the oral mucosa to cell immune responses. The activation of CD4+ T- and CD8+ T-lymphocytes, followed by the ejection of cytokines, such as interleukin-2 (IL-2), interferon-gamma (IFN-g), and tumor necrosis factor (TNF), leads to the apoptosis of keratinocytes [1,2,3,4,5].

LP may affect the skin, scalp, hair follicles, nails, and oral and genital mucosa. Rarely, it may also involve the eyes, urinary tract, nasal mucosa, and larynx [1,2]. Typical skin presentation includes itchy, purple, flat papules. At the same time, characteristic oral findings cover the presence of multiple keratotic white papules arranged in reticular or plaque-like networks with a bilateral and symmetric distribution. Less commonly erosive, erythematous, or bullous lesions may also develop. Therefore, oral lichen planus (OLP) can be classified as reticular, plaque, papular, erosive, atrophic, and bullous. All types of OLP can be pooled into two main clinical groups: erosive-atrophic forms (EA-OLP), including erosive, atrophic, bullous, and mixed EA variants, and non-erosive-atrophic forms (non-EA-OLP), comprising of reticular, plaque, and papular subtypes [1,3,6,7,8]. A common phenomenon accompanying OLP is desquamative gingivitis [9,10,11]. Skin and mucosal lesions may develop both alone or together, as well as simultaneously or independently. The reported coincidence rate is inconsistent depending on the authors, varying from 4% to 44%. It has commonly been considered that approximately one-third of patients presenting oral lesions also present skin lesions [2,11]. The oral form of lichen planus occurs more frequently and tends to be more resistant to treatment than the cutaneous form.

Upon histopathological examination, most characteristic findings in the OLP include lichenification of the basement layer, followed by a marked layered lymphocytic infiltrate underlying the epithelium, the presence of numerous eosinophilic colloid bodies along the epithelial-connective tissue interface (Civatte bodies), sawtooth-shaped interpapillary ridges, variable thickness of the spinous layer, and the presence of ortho- or parakeratosis [2,5,7]

The OLP prevalence in the general adult population is estimated as 0.5 to 2% [2,12] with a female predilection (female: male ratio 2:1) and the age of onset between 30 and 60 years [7,9]. OLP in children has been observed only in exceptional cases [1,2].

OLP is classified as an oral potentially malignant disorder (OPMD) by the World Health Organization (WHO), with a transformation risk from 0.4% to 3.3%, over a period 0.5 to >20 years, more commonly deriving from atrophic-erosive types [8,11]. In the recent meta-analysis by Idrees et al., the average risk of developing a malignant disorder in patients with OLP was calculated at 1.37%, which is low [13]. The exact mechanisms of carcinogenesis in OLP have still not been fully understood. However, some components of oral virobiota have been suggested as stimulants of the OLP development and progression from non-EA into EA types, namely hepatitis C virus (HCV), Epstein–Barr virus, herpesvirus (HSV), cytomegalovirus (CMV), and human papillomavirus (HPV) [2,7,14,15]. Although it has not been well defined whether these factors are genuinely causative for OLP or only superimpose the already existing autoimmune disease, several meta-analyses have demonstrated strong evidence of an association between viral infections and OLP, especially regarding HCV [16,17]. This agent can bind cells other than hepatocytes, including epidermal cells. The repeated activation of the immune cells due to the viruses’ high mutation rate may lead to the dysregulation of the immune system and trigger the autoimmune response [5].

HPV has also been considered a potential trigger for the malignant transformation of the OLP, with the first reports on the association between HPV and OLP dated back to 1987 [18]. HPV is a small, non-enveloped virus with a diameter of 52–55 nm. Its genome contains a double-stranded DNA molecule bound to cellular histone [19] that encodes approximately eight open reading frames (ORFs), showing three functional parts as follows: the early (E) region, the late (L) region, and a long control region (LCR). Proteins called E1, E2, E4, E5, E6, and E7, which participate in the replication, cellular transformation, and control of viral transcription, are encoded by the E region [19,20].

The oncogenic activity of HPV in the anogenital region and oropharynx has already been well established; in particular, HPV infection is recognized as a decisive risk factor for high-grade squamous intraepithelial lesions (HSILs) and invasive cervical cancers. Simultaneously, HPV has a strong affinity for squamous epithelial cells, and several virus genotypes are responsible for developing benign mucosal lesions, like warts, squamous papillomas, and condylomas, or installing a latent infection in the basal keratinocytes. The course of HPV infection and the risk of malignant transformation of the lesions is determined by the type of virus, modified by various physical, chemical, and biological agents, the genetic constitution, and the immune defense mechanisms of the host [1,21]. A division of the virus oncogenic phenotypes into high- and low-risk types is based on the differences in the DNA base sequences of E6 and E7 genes. High-risk types include HPV 16, 18, 31, 33, 35, 45, 51, 52, 56, 58, and 59, while the low-risk include HPV 6, 11, 42, 43, and 44 [19,20]. The International Agency for Research on Cancer (IARC) classified HPVs into three groups according to their oncogenic activity. Groups 1 and 2 are considered high-risk genotypes, as they tend to incorporate their DNA in the host cell genome rather than keeping a minimal transcriptional activity in an episomal form. The high-risk types of HPV 16 and 18 are closely associated with squamous cell carcinomas and have been detected with a rising frequency in precancerous lesions of increasing grades in the cervix uteri [21]. The predominant genotypes in benign genital lesions, rarely found in carcinomas, are HPV 6 and 11. The effects of high-risk HPV types on anogenital mucosal membranes are explained by the capacity of HPV 16 and 18 to compromise physiological cell cycle control by continuous expression of their E6 and E7 genes and binding of the related oncoproteins to the tumor suppressor gene products p53 and pRB [22,23]. The explicit confirmation that this mechanism also refers to the oral region still needs to be improved. Many studies confirmed the presence of different types of HPV in oral cancer, especially using PCR-based DNA detection techniques, showing a wide range of HPV detection rates [21,22,23,24,25]. A few authors have also described the studies on HPV detection in OPMD, presenting conflicting results [24,26,27,28,29]. Therefore, we believe that performing the meta-analysis to evaluate the association of HPV in OLP is justified and needed.

This meta-analysis aimed to determine the potential association between HPV and OLP based on case–control and cross-sectional study results.

## 2. Results

### 2.1. Overall Information on the Studies Included

**Figure 1:**
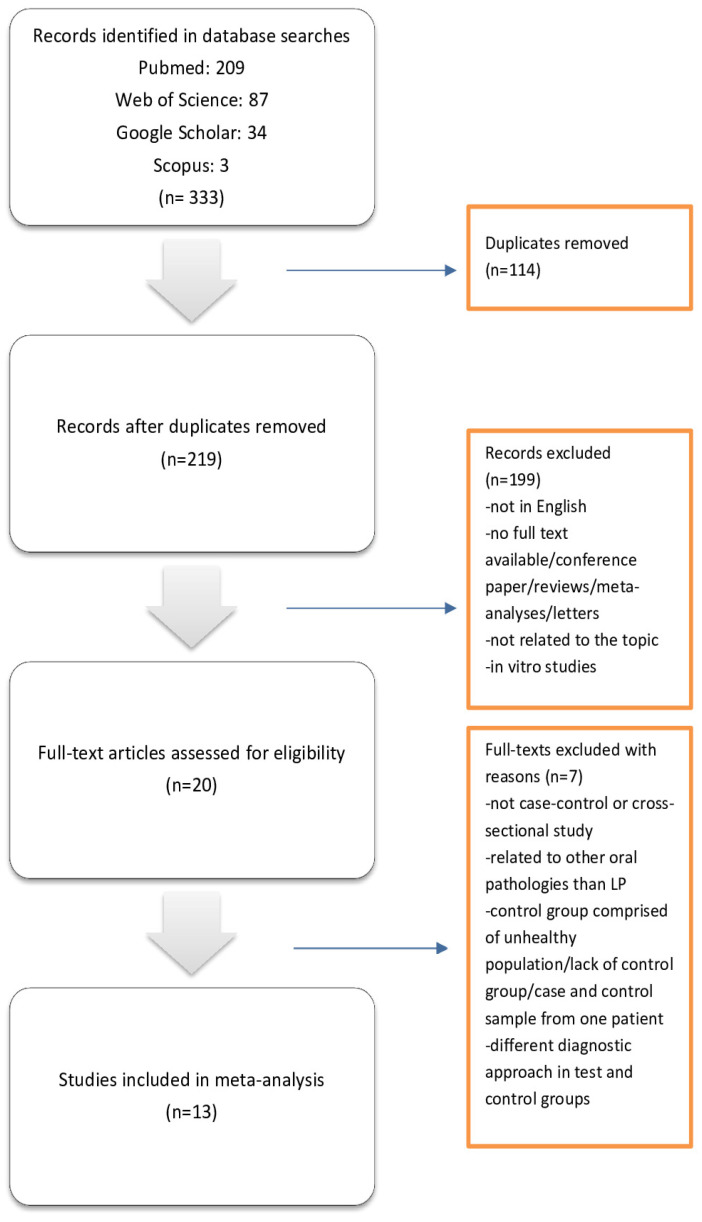
Search strategy according to PRISMA guidelines.

The search strategy is presented in Figure 1. From the 333 articles identified through initial research, 114 were excluded as duplicates. Further, 199 were excluded for lack of relevance to OLP and HPV, papers not in English, papers with no full text available, conference papers/reviews/meta-analyses/letters, or in vitro studies. From the remaining 20 papers, seven were excluded for reasons such as presenting no case–control or cross-sectional study, being related to other oral pathologies than LP, having a control group comprised of an unhealthy population/lack of control group/case, and having a control sample from one patient or using the different diagnostic approach in test and control groups. Finally, thirteen papers met the inclusion criteria of this study.

The details of the selected studies are listed in Table 1. Studies on OLP included 541 cases, of whom 219 were HPV-positive, and 413 controls, of whom 56 were HPV-positive. The analysis covered eight countries: three from Asia (India [28,30], Iran [6,31,32], and Turkey [15]), and five from Europe (Czech Republic [33], Hungary [34], Italy [35,36], Macedonia [37], and the UK [18]). In 7 of the 13 included papers, the differentiation into the erosive-atrophic type and non-erosive-atrophic type of OLP was utilized. The tested HPV genotypes included: 6, 11, 16, 18, 26, 31, 33, 35, 39, 40, 42, 45, 51–56, 58, 59, 61, 62, 64, 66–73, 81–84, IS39, and CP6108. In the majority of studies, the RT-PCR method was utilized to detect HPV, while immunohistochemical staining was performed in two studies only [14,28]. To collect the biological material, a brush-biopsy technique was used in three studies [35,36,37], and incisional biopsy in one [18]. Formalin-fixed paraffin-embedded tissue specimens were analyzed in the remaining nine studies. In those studies, the diagnosis of OLP was confirmed histologically, as it was in the Campisi et al. study [35]. There was no information on such a confirmation of the OLP diagnosis in the study groups in the other cited articles [36,37]. Only in three studies were the cases and controls age-matched [33,34,38], while in none of the studies were they sex-matched.

### 2.2. Quality Assessment

Based on the criteria described in the NOS manual, all the included studies presented high quality (scores between 7 and 9), except one study awarded 6 stars [31] (Table 2). Please note that for some factors of the NOS scale, more comments are required. In the comparability of cases and controls in one study [31], differences between subgroups were statistically significant (only one case of virus in control), but no correction was applied. In another case [38], only a similar age group was mentioned, and the study design did not introduce the exact age match. However, we have still decided to award the study one star. In another case [30], the authors could not calculate the statistical significance of age and, for other statistically significant factors, no correction was applied. Finally, in one publication [37], only percentages were mentioned for potentially confounding factors, but no statistical tests and corrections were performed. We awarded all publications with one star in case of a non-response rate, as the samples were collected once. Therefore, there was no possibility of non-returning/non-responding patients.

### 2.3. Meta-Analysis

Egger’s test value for the mixed-effects meta-regression model was z = 3.2631, *p* = 0.0011, meaning that part of the results that were not satisfactory might have not been published. Therefore, we decided to use a trim-and-fill method, allowing the prediction of the missing publications. In brief, this method predicted that six studies needed to be simulated to properly represent the missing data. That was almost half of the collected data, which shows that the trim-and-fill method is also not a perfect solution. Therefore, we present the remaining results for both original and filled/trimmed data.

**Figure 2:**
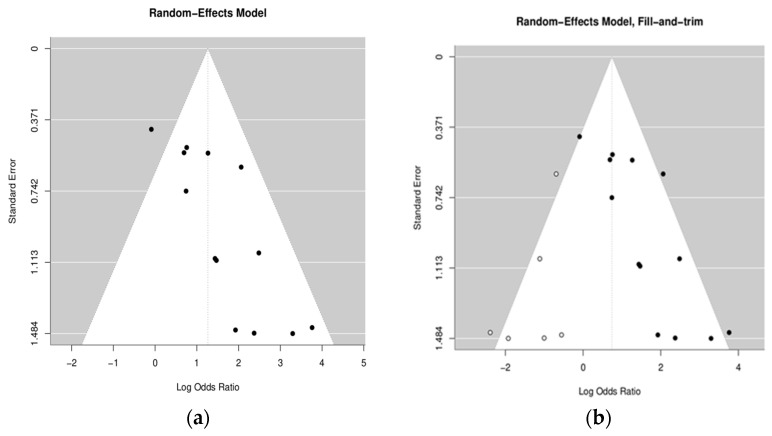
Funnel plots without (**a**) and with (**b**) simulated publications.

Funnel plots without and with simulated publications are presented on Figure 2.

Table 3 ilustrates heterogeneity data for both raw data and data after the fill and trim procedure.

Chi^2^ test results were as follows: estimated value 1.2651, standard error 0.2888, z-value: 4.38, *p*-value < 0.0001 for raw data, and an estimated value 0.7425, standard error 0.3051, z-value 2.4340, and a *p*-value 0.0149 for fill and trim data. OR values were 3.54 [2.01, 6.24] and 2.10 [1.16, 3.82], respectively. These values suggest that there is at least a two times bigger chance for a person with HPV to have OLP.

**Figure 3:**
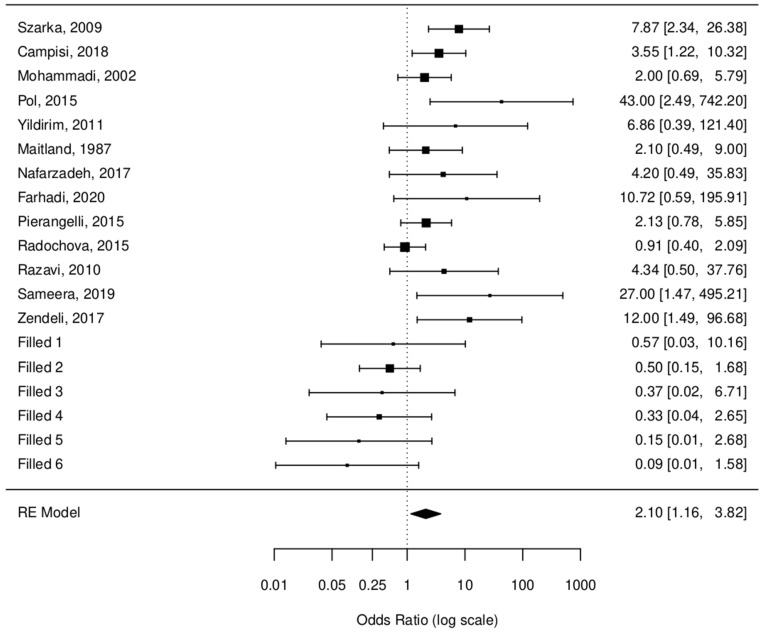
Random effect model [6,15,18,28,30,31,32,33,34,35,36,37,38].

Figure 3 shows the random effect model.

To check sensitivity, we utilized the “leave one out” method. In this method, statistics are calculated multiple times for data where one of the publications is excluded. Thanks to this approach, we were able to see that elimination of any single publication from our study did not drastically change the predictions. In all cases, the results of the meta-analysis were significant, meanwhile one publication [33] seemed to noticeably influence heterogeneity.

Table 4 presents the results of sensitivity test using “leave one out” method.

## 3. Discussion

This meta-analysis was performed to identify the association between HPV and OLP and revealed that HPV infection was associated with at least a two times higher risk for a person with HPV to have OLP, depending on whether the original data or filled/trimmed data were used. This stands by some previously published studies that showed even a higher OR than ours [3,19,25]. The association between HPV infection and OLP, but also other potentially malignant oral disorders, has been an object of several studies; however, the exact mechanism of viral interaction with chronically inflamed oral mucosa, as it occurs in OLP, has not been efficiently explored so far. HPVs present a unique tropism for squamous keratinocytes. After the integration of viral and host genomes, the death of keratinocytes occurs [39]. The epithelial thinning observed in atrophic-erosive forms of OLP facilitates the virus adhesion to the basal cells. On the one hand, it can be assumed that epithelial damage enhances microbial colonization. HPV penetrates the epithelium via micro-lesions and gaps, while highly keratinized regions become colonized less frequently. On the other hand, the dysregulation of the immune system, which is one of the etiopathologic mechanisms of OLP, may also promote viral growth. From this point of view, HPV infection presents as secondary to OLP [11].

Additionally, steroids commonly used in the treatment of “red types” of OLP result in immunosuppression, which can be followed by the upregulation of HPV expression [25,40]. Decreased immune cell concentration and reduced cytokine production, which are induced by steroidal treatment, may influence the HPV transcription and enhance the extracellular HPV persistence in the epithelium to accomplish its replication cycle [40,41]. patients. In our search, we did not find any results of the research on the prevalence of HPV in OLP before and after corticosteroid therapy. This issue was also pointed in a Agha-Hosseini and Hafezi Motlagh review, who emphasized the necessity to extend studies in this field [9].

By altering the host cell activity and promoting the expression of abnormal proteins, HPV could potentially support the progression of lichen planus, and the persistence of the viral infection could also explain the chronicity of the condition. HPV infection creates a pro-inflammatory environment by the increase of cytokines, and by stimulating cellular molecular pathways involving TLR-4 and NF-KB. Thus, the virus presence in autoimmune disease could concur to induce oncogenesis [11].

Moreover, persistent viral infections lead to immune dysregulation and promote the slow progression from infection to dysplasia and cancer [41]. HPV 16 and 18 compromise the physiological control of the cell cycle by continuous expression of E6 and E7 genes and by binding the related oncoproteins to the tumor suppressor gene products p53 and pRB in anogenital tumors, especially carcinomas of the cervix uteri [23]. However, the importance of these basic mechanisms in oral carcinogenesis has not yet been entirely understood; a causal action of HPV in oral cancerogenesis remains not fully established.

A linkage between HPV infection, OLP, and risk of malignant transformation via p16 gene expression was suggested in the study by Liu et al. [39]. The authors demonstrated that p16 and HPV16/18 (E6) expression increased in OLP tissue and malignantly transformed OLP compared to the controls. Therefore, the expression of p16 could act as a marker to predict HPV16/18 infection in OLP, while the HPV16/18 infection might contribute to the malignant transformation of OLP [39]. Epithelial carcinogenesis, characterized by uncontrolled cellular growth, is initiated by gene mutation and protein expression alterations of the corresponding gene. That leads to morphological changes, which do not always allow for predicting a possible carcinoma progression [19,20,39]. Molecular markers, like p16, could potentially facilitate that.

According to some authors, HPV16 detection in the mouth is strongly associated with HPV16 persistence in the genital tract and, therefore, may also lead to the progression of cervical cancer. These studies suggest that the oral region may act as a natural reservoir of HPV at a locus outside of the genital region, and potentially become a reinfection focus [42,43] as HPV16 can integrate with host-cell DNA and activate oncogenes. The interaction of various oral microorganisms and their adhesion results in the formation of a microbial community through aggregation and coaggregation. The oral dysbiosis and synergistic effects in oral microbial communities may promote cancer development [43]. The persistence of the HPV infection in the oral area is more frequently associated with high-risk HPV genotypes and is the major risk factor for the oral dysplasia [43].

As the data for this publication were biased, we used the “fill and trim” method. However, it introduces extra heterogeneity (I^2^) and should be treated cautiously. Both approaches (with and without the fill and trim method) suggest a correlation between OLP and HPV. However, the calculated odd ratio values differ, as estimated missing publications reduced this value.

This meta-analysis has a few limitations. As the data were insufficient or the number of the examined samples was too low to perform a subgroup analysis, we did not compare the impact of the specific HPV genotypes on the OLP occurrence. For the same reasons, the anatomic location of the oral lesions and clinical subtype of the OLP were not analyzed in correlation with the HPV status. This remains accordant with the observations of Agha-Hosseini and Hafezi Motlagh, who also concluded that the prevalence of HPV in OLP has not been widely discussed in terms of the clinical type of OLP lesions, and further investigation of this topic is definitely required [9]. The methods for the sample collection and the HPV detection used in the included studies were not uniform, which partially results from the wide timeframe of the described research, ranging from 1987 to 2023. However, up to now, a gold standard for the HPV detection in the oral lesions has not been established. Another limitation that needs to be considered is that in most of the studies, cases and controls where not age- and sex-matched. The detection, diagnostic, and selection bias of the included case–control studies could have also influenced the results of this meta-analysis.

## 4. Materials and Methods

### 4.1. Search Strategy

A systematic search was performed in PubMed (www.ncbi.nlm.nih.gov/pubmed accessed on 6 March 2024), Web of Science (SCI), Google Scholar, and Scopus databases with the last update on 6 March 2024, with the following search terms: (lichen planus or LP or oral lichen planus or OLP) and (human papillomavirus or HPV), and Web of Science category “Dentistry, Oral Surgery & Medicine”. The references of the selected papers were additionally sifted through for potentially eligible studies. No time limits for the publication date were used in the search criteria.

Two investigators (ZŚ and MLW) performed the literature search independently; in case of disagreements, a consensus was reached in consultation with a third investigator (TW). The reviewers were not blinded to the studies’ authorship. Initially, the records were assessed by the investigators according to the relevance of the title or the abstract. When the information determining if a study fulfilled the inclusion criteria was insufficient, the same reviewers assessed the full report. The same two reviewers verified the full texts of the studies considered potentially eligible by at least one of the investigators in the initial search.

The following inclusion criteria were used for the papers enrolled in this meta-analysis: to investigate the presence of HPV in patients with OLP, to describe an original, case–control, or cross-sectional study with a full-text available in English. Studies where the controls were suffering from any oral disease other than OLP, the studies where the case and control samples were collected from the same person, the studies where a different diagnostic approach was utilized in cases and controls, and the studies with insufficient data, were excluded from this meta-analysis. We also excluded case reports, review papers, and meta-analyses, papers where no full text was available and those that were published in a language other than English.

The same two investigators independently extracted data from each study included in this review. The following details were collected: authors, year of publication, country of origin, study design, clinical type of OLP, number of OLP patients and healthy controls, test methods, HPV genotypes, and the % of OLP patients and healthy controls positive for HPV, including the division into OLP subtypes (if given).

Statistics were calculated using the metafor package for R (version 4.4-0, R version 4.3.2) [44]. The R code was deployed on github: https://github.com/tomaszwozniakihg/metaanalysis_olp_hpv, accessed on 21 June 2024.

### 4.2. Quality Assessment

The Newcastle–Ottawa Scale (NOS) for case–control studies was used to analyze the quality of publications. The scale evaluates three quality parameters (selection, comparability, and outcome) divided across eight specific items. Each item on the scale is scored from one point, except for comparability, which can be adapted to the topic of interest to score up to two points. The maximum score for each study was 9, with less than 5 points being identified as representing a high risk of bias [45].

## 5. Conclusions

In this meta-analysis, we found OLP to be strongly associated with HPV. Further prospective cohort studies with a uniform research standards are required to understand the role of HPV as an etiological factor of OLP.

## Figures and Tables

**Table 1:** General characteristics of the studies included in the meta-analysis.

Author	Country	Cases (LP)	Controls	HPV+ Cases	HPV+ Controls	Types of HPV Tested	Type of LP	HPV+ Cases/LP Type
Campisi G. et al. (2004) [35]	Italy	71	90	14	5	6, 16, 18, 31, 33	AE/n-AE	AE HPV+ 9 n-AE HPV+ 5
Farhadi S. et al. (2020) [38]	Iran	32	20	8	0	11, 16, 18, 33–73	AE/n-AE	AE HPV+ 8 n-AE+ 0
Maitland NJ. et al. (1987) [18]	UK	8	12	7	5	16	NA	NA
Mohhamadi M. et al. (2023) [6]	Iran	25	25	14	7	16, 18	AE/n-AE	AE HPV+ 8 n-AE HPV+ 6
Nafarzadeh S. et al. (2017) [31]	Iran	50	7	30	1	NA	NA	NA
Pierangeli A. et al. (2016) [36]	Italy	12	54	9	19	6, 11, 16, 18, 31, 33, 53, 58	NA	NA
Pol CA. et al. (2015) [28]	India	30	30	21	0	16	n-AE	n-AE HPV+ 21
Razavi SM. et al. (2009) [32]	Iran	29	9	14	1	18	NA	NA
Radochova V. et al. (2015) [33]	Czech Republic	45	24	24	14	6, 11, 16, 18, 26, 31, 33, 35, 39, 40, 42, 45, 51–56, 58, 59, 61, 62, 64, 66–73, 81–84, IS39, CP6108	AE/n-AE	AE HPV+ 16 n-AE HPV+ 8
Sameera A. et al. (2015) [30]	India	15	15	13	0	18	AE/n-AE	AE HPV+ 5 n-AE HPV+ 8
Szarka K. et al. (2009) [34]	Hungary	119	72	39	3	6, 11, 16, 18, 31, 33	AE/n-AE	AE HPV+ 26 n-AE HPV+ 13
Yildirim B. et al. (2012) [15]	Turkey	65	15	14	0	16	NA	NA
Zendeli-Bedejeti L. et al. (2019) [37]	Macedonia	40	40	12	1	16, 18, 31, 33, 35, 39, 45, 51, 52, 56–59	NA	NA

AE: atrophic-erosive; n-AE: non-atrophic-erosive; NA: not applied.

**Table 2:** Assessment of quality of the studied included in the meta-analysis with the NOS scale.

Reference	Selection				Comparability		Exposure			Total
	1	2	3	4	1 (Age)	2 (Other)	1	2	3	
Campisi G. et al. (2004) [35]	*	*	*	*	*	*	*	*	*	9
Farhadi S. et al. (2020) [38]	*	*	*	*	* ^1^	0	*	*	*	8 ^1^
Maitland NJ. et al. (1987) [18]	*	*	*	*	0	0	*	*	*	7
Mohhamadi M. et al. (2023) [6]	*	*	*	*	*	*	*	*	*	9
Nafarzadeh S. et al. (2017) [31]	*	*	0	*	0 ^1^	0 ^1^	*	*	*	6 ^1^
Pierangeli A. et al. (2016) [36]	*	*	*	*	*	*	*	*	*	9
Pol CA. et al. (2015) [28]	*	*	*	*	*	*	*	*	*	9
Razavi SM. et al. (2009) [32]	*	*	*	*	0	*	*	*	*	8
Radochova V. et al. (2015) [33]	*	*	*	*	*	*	*	*	*	9
Sameera A. et al. (2015) [30]	*	*	*	*	0	0 ^1^	*	*	*	7 ^1^
Szarka K. et al. (2009) [34]	*	*	*	*	*	0	*	*	*	8
Yildirim B. et al. (2012) [15]	*	*	*	*	0	0	*	*	*	7
Zendeli-Bedejeti L. et al. (2019) [37]	*	*	*	*	0 ^1^	0	*	*	*	7 ^1^

*—1 point (1 star); ^1^—justification of the evaluation is presented in the paragraph above.

**Table 3:** Heterogeneity data for both raw data and data after the fill and trim procedure.

	Raw Data	Fill and Trim
Tau^2^	0.3937	0.8279
I^2^	41.62%	55.74%
H^2^	1.71	2.26
Test for heterogeneity		
Q	20.1027	40.3356
*p*-val	0.0652	0.0019

**Table 4:** Sensitivity test results using the “leave one out” method.

	Estimate	Standard Error	z-Val	*p*-Val	ci.lb	ci.ub	Q	Qp	Tau^2^	I^2^	H^2^
Szarka K. et al. (2009) [34]	1.14	0.29	3.8885	0.0001	0.57	1.71	16.93	0.11	0.33	36.02	1.56
Campisi G. et al. (2004) [35]	1.31	0.33	3.9855	0.0001	0.66	1.95	19.87	0.05	0.51	46.09	1.85
Mohhamadi M. et al. (2023) [6]	1.38	0.32	4.2424	0.0000	0.74	2.01	19.68	0.05	0.49	45.01	1.82
Pol CA. et al. (2015) [28]	1.15	0.28	4.1410	0.0000	0.61	1.70	16.48	0.12	0.31	37.47	1.60
Yildirim B. et al. (2012) [15]	1.25	0.30	4.2133	0.0000	0.67	1.83	19.72	0.05	0.41	43.94	1.78
Maitland NJ. et al. (1987) [18]	1.35	0.32	4.2361	0.0000	0.72	1.97	19.95	0.05	0.48	46.58	1.87
Nafarzadeh S. et al. (2017) [31]	1.27	0.30	4.1718	0.0000	0.67	1.87	19.96	0.05	0.44	45.38	1.83
Farhadi S. et al. (2020) [38]	1.23	0.29	4.1908	0.0000	0.65	1.80	19.26	0.06	0.39	42.84	1.75
Pierangeli A. et al. (2016) [36]	1.37	0.33	4.2042	0.0000	0.73	2.01	19.79	0.05	0.50	45.08	1.82
Radochova V. et al. (2015) [33]	1.37	0.24	5.7578	0.0000	0.90	1.83	11.07	0.44	0.04	5.25	1.06
Razavi SM. et al. (2009) [32]	1.27	0.30	4.1703	0.0000	0.67	1.87	19.93	0.05	0.44	45.28	1.83
Sameera A. et al. (2015) [30]	1.18	0.28	4.1563	0.0000	0.62	1.74	17.71	0.09	0.34	39.76	1.66
Zendeli-Bedejeti L. et al. (2019) [37]	1.18	0.29	4.0781	0.0000	0.61	1.75	18.15	0.08	0.35	39.95	1.67
All (none removed)	1.27	0.29	4.3800	0.0001	0.70	1.83	20.10	0.07	0.39	41.62	1.71

## Data Availability

The data presented in this study are available on request from the corresponding author.
